# Therapeutic Effect of Epidermal Growth Factor Combined With Nano Silver Dressing on Diabetic Foot Patients

**DOI:** 10.3389/fphar.2021.627098

**Published:** 2021-04-23

**Authors:** Kaihua Zhang, Yiran Li, Jiarui He, Jiasheng Xu, Yanhua Wan, Shasha Wan, Riwei Wang, Qingfu Zeng

**Affiliations:** ^1^ Department of Vascular Surgery, The Second Affiliated Hospital of Nanchang University, Nanchang, China; ^2^ Department of General Surgery, The Jiujiang Affiliated Hospital of Nanchang University and Jiujiang First People’s Hospital, Nanchang, China; ^3^ Queen Mary College, Nanchang University, Nanchang, China; ^4^ Department of Clinical Laboratory, The Second Affiliated Hospital of Nanchang University, Nanchang, China

**Keywords:** diabetic foot, epidermal growth factor, nano silver dressing, wound healing, therapeutic effect

## Abstract

**Objective:** To investigate the clinical efficacy of epidermal growth factor combined with nano silver dressing in the treatment of diabetic foot wounds.

**Methods:** A total of 160 patients with diabetic foot ulcers admitted to the Second Affiliated Hospital of Nanchang University from 2015-06 to 2018-06 were selected to participate in the experiment. A randomized table method was used to randomly divide 160 patients into 4 groups: 40 in the epidermal growth factor group, 40 in the nano-silver dressing group, 40 in the combined group, and 40 in the saline control group (normal saline). The healing stage of the wound surface and the growth degree of granulation tissue were graded. Each group was given a dressing change every other day, and the time required for wound repairing to each healing stage was observed. After 2 and 4 weeks of treatment, the wound exudate was collected for bacterial culture.

**Results:** There was no significant difference in the time between the four groups of patients reaching the effective phase of treatment (level 1). Compared with the control group, the epidermal growth factor group and the combined group achieved a shorter time for wound repairing to healing stages 2 and 3, and the difference was significant (*p* < 0.05). The combined group had a shorter wound repairing time than the epidermal growth factor group (*p* < 0.05). Compared with the control group, the positive rate of bacteria in the combined group and the silver nanoparticles group was significantly lower after 2 and 4 weeks of treatment.

**Conclusion:** There is no significant difference in wound healing between the four groups during the clinically effective period. After this period, the combined use of recombinant epidermis Growth factors and nano-silver dressings have a significant effect on promoting wound healing and can effectively prevent infection.

## Introduction

Diabetic foot is a serious diabetic complication (Huang et al., 2015) with high clinical incidence which can easily cause disability and seriously affect the life and health of patients. Diabetic patients have symptoms of infection in the lower limbs due to autonomic dysfunction and vascular disease, and the affected limb can form ulcers and damage to soft tissues of the skin. The conventional treatment methods for diabetic foot mainly include debridement and drainage, treatment with antibacterial drugs, and control of blood sugar. Through conventional treatment, the amputation rate of diabetic foot is reduced to a certain extent, but there are still many diabetic foot patients whose wounds are difficult to heal and the incidence of wound infection is high (Li et al., 2016; Zhang et al., 2016), so it is necessary to explore a more reasonable treatment plan. In recent years, with the deepening of biomolecular technology research, scientists have found that growth factors have an important role in promoting wound repair. Therefore, epidermal growth factor (EGF) is increasingly used in the treatment of refractory wounds to rebuild blood vessels and promote epidermal cell proliferation. Nanosilver is a particle processed based on nanotechnology, which gives it a strong skin permeability and good stability due to its unique process. Covering the nano-silver on the medical gauze, the reticular fiber structure can be realized, which is beneficial to the exudation and drainage of the wound surface, and can also exert the heavy metal characteristics of the silver, causing the activity of the protein to be reduced, thereby achieving the sterilization effect. Due to the convenient use of nano silver dressings, the antibacterial effect is reliable, and it is widely used in the treatment of refractory wounds such as pressure sores and burns. Epidermal growth factor has been widely used in the clinical treatment of diabetic foot, but there are very few clinical studies on the treatment of epidermal growth factor combined with nano silver dressing. This study designed a control experiment to observe the effect of epidermal growth factor and nano-silver dressings on the treatment of diabetic foot alone and to compare the therapeutic effects after the combination of the two, to determine the therapeutic effect of diabetic foot wounds treated with epidermal growth factor combined with nano-silver dressing.

## Materials and Methods

### General Information

160 patients with diabetic foot in our hospital from July 2015 to July 2018 were selected. The patients were randomized into four groups by digital table method. All patients signed the informed consent and volunteered to participate in the study. The study was approved by the ethics committee of the Second Affiliated Hospital of Nanchang University. There were 160 patients in the observation group, including 83 males and 77 females, aged 51–73 years old, with an average of (58 ± 2.6) years, a history of diabetic foot was 1–6 years, with an average of (3.25 ± 0.64) years.

### Clinical Inclusion and Exclusion Criteria

Inclusion criteria: 1) All patients met the diagnostic criteria for diabetic foot established by the World Health Organization (WHO) and the American Diabetes Association (ADA), that is, ① + any of ②③④: ①Diagnosis of type 12 diabetes is clear; ② The dorsal artery pulsation weakened or disappeared, the foot was ischemic, the skin was pale and dark, and the affected foot had ant feeling; ③ the affected foot had blisters, ulcers or gangrene.④ The affected foot had neuropathy such as loss of temperature sensation, increased pain, and muscle atrophy. 2) The patients in this study were all eligible for diabetic foot II and III patients diagnosed by Wagner classification (Yang et al., 2014; Ju, 2015; Mao et al., 2015). 3) The wound was located in the foot and the blood perfusion was good (or the wound was located at the toe, but the patient required limb salvage treatment). 4) Patients who participated in the study were informed and voluntarily signed informed consent, which was approved by the hospital ethics committee.

Exclusion criteria: exclude patients with severe vascular circulatory dysfunction; those who are allergic to drugs or dressings; exclude patients with cardiovascular diseases such as coronary heart disease, severe hypertension [patients with hypertension have one of the following: 1) After medication, blood pressure remains high or unstable; 2) First diagnosed with hypertension but unwilling to receive medication], severe liver and kidney damage, and patients with neurological disorders.

### Methods

After admission to all hospitals, the physician should carefully observe the patient’s foot wound. For patients who had been diagnosed with diabetes, the patient should be asked about the type of clinical medication, the number of medications, and blood glucose control, the patient’s blood glucose profile should be measured, and the clinical medication adjusted in time. All patients received clinical education on diet control, which caused patients to pay attention to their own blood sugar. All the patients in each group controlled the fasting blood glucose below 11.1 mmol/L before the treatment of the wound, and the patients with the infection were treated with the corresponding antibiotics until the infection was completely controlled (the comparative study was started 3 days after the antibiotic was stopped), and the wound was debrideed, cleaned, and the pathological granulation was removed. The experimental design, intervention implementation, and evaluation were all carried out by our team, and all the team members had received relevant professional training. For patients with deep tissue damage and soft tissue inflammation, the physician should perform debridement according to the requirements of aseptic operation, remove the necrotic tissue at the foot ulcer of the patient, and open the drainage as soon as possible for the patient who has formed the abscess or sinus. The extract was subjected to bacterial culture and drug sensitivity tests.

#### Grouping

A randomized grouping method was used to randomly divide 160 patients into 4 groups: 40 patients in the combination group, 40 in the epidermal growth factor group, 40 in the nano-silver dressing group, and 40 in the control group.

#### Treatment

According to the recommended dosage of the drug label, the combination group was given JinYintai (Shenzhen Huashengyuan Gene Engineering Development Co., Ltd., S20010038) (exogenous recombinant human epidermal growth factor) 40 IU/cm^2^ sprayed evenly on the wound surface. The nano silver dressing (Paul Hartmann AG, import product registration standard: GEM 1735-2010) was used for dressing. For patients with foot ulcers which had formed sinus tracts, doctors should first spray JinYintai evenly on nano silver dressing, then make the dressing into strips to be stuffed into the sinus tract, and the rest of the treatment was the same as that of the control group. The epidermal growth factor group was given JinYintai (exogenous recombinant human epidermal growth factor) 40 IU/cm^2^ each time, evenly sprayed on the wound surface, and the other treatments were the same as the control group; the nano silver dressing group was replaced with nano silver dressing each time; The control group was treated with common surgical dressing (disinfection, debridement, and drainage), and the wound was wiped with physiological saline (The gauze was soaked in physiological saline, and the gauze was applied to the wound surface, mainly for wound moisturizing and astringent wounding effect). Each group was given a dressing change every other day. The methods of quantitative analysis of percent of healing values: The two people in our group measured and checked at a time set. Measurement method: using Vernier caliper to the longest edge of wound, as reference scale. Then pictures were taken directly above the wound, and pixels were set to 300 pixels per inch by Photoshop software. The Vernier caliper scale and wound range were drawn along the boundary by using the “polygon lasso tool” and Eclosion was set to 0. Then the image size of Vernier caliper can be obtained by selecting all channel views in the window histogram interface, so as to calculate the proportion of the picture and actual size, and then calculate the wound area proportionally. More accurate area measurement values can be obtained. For wounds located on curved surfaces, by taking pictures from multiple angles at the top of the wound, the wound area was measured and evaluated in different pictures.

### Observation Indicators

The time required for wound healing to each healing stage was observed, and the healing rate of the wound surface and the growth degree of granulation tissue were graded in the healing stage.

**FIGURE 1:**
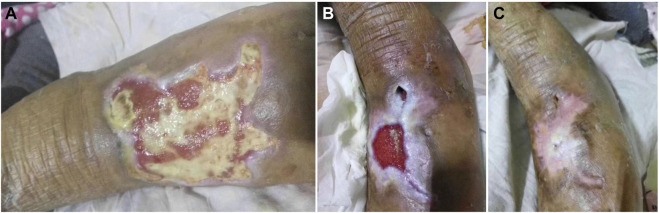
Clinical reference pictures: **(A)**: the wound healing reaches 40% of the total wound **(B)**: the wound healing reaches 70% of the total wound **(C)**: the wound healing reaches 100% of the total wound.

**FIGURE 2:**
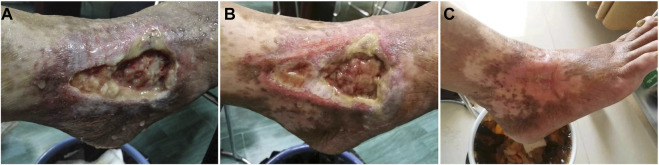
Clinical reference pictures: **(A)**: wound granulation tissue formation accounted for 40% of the total wound volume **(B)**: wound granulation tissue formation accounted for 70% of the total wound volume **(C)**: wound granulation tissue formation accounted for 100% of the total wound volume.

Wound healing rate = (initial area of wound - corresponding time point area)/initial area of wound * 100% Classification of granulation maturity: The growth of granulation tissue in each group was observed and described. According to the “Guidelines for Clinical Research of New Drugs for Treatment of Acute Sores,” the efficacy criteria in this study were determined as follows (Song et al., 2014):

Healing level 1: wound healing rate of 40% (Figure 1), or wound granulation tissue formation accounted for 40% of the total wound volume (Figure 2). Healing level 2: wound healing rate of 70% (Figure 1), or wound granulation tissue formation accounts for 70% of the total wound volume (Figure 2). Stage 3 of healing: the wound healing rate is 100% (Figure 1), or the granulation tissue of the wound completely covers the wound (Figure 2).

The time required for each group of wounds and granulation tissue repair to reach 1, 2, and 3 levels was recorded. After 2, 4 weeks of treatment, the wound exudate was cultured, and the bacterial culture results of the wound exudate of each group were recorded. The positive rate of bacterial culture was calculated and compared between groups. When the patients reached each healing stage, the wound exudate was collected for bacterial count and the difference between the four groups was compared (At stage 3, the wound was cleaned with saline if there was not enough exudate for sampling).

### Statistical Methods

Statistical analysis data were performed using SPSS18.0 system software; the measurement data were expressed by (x ± s) and *t* test; the count data was expressed by [*n* (%)], and the χ2 test was used; *p* < 0.05 indicated that the difference was statistically significant. SPSS software was used to determine whether the data conform to the normal distribution by Shapiro-Wilk Test. The basic data of each group of patients were analyzed by ANOVA to test whether there was any difference, and the analysis results were verified by Tukey post hoc tests.

## Results

### Basic Information of Participants and Comparison of Differences Between Groups

According to the inclusion and exclusion criteria, 160 patients with diabetic foot who met the inclusion criteria were included in the study. There was no exclusion during the study, and all the results were analyzed. The variance analysis was performed in 4 groups of patients with age, diabetic foot history, original wound size, and wound formation time. There was no significant difference in basic information between the groups (*p* > 0.05), which could be compared and analyzed. For general baseline data for each group, see Table 1 **.**

**TABLE 1:** Comparison the basic information of 4 groups.

Group	Gender		History of diabetic foot (year)	Age (years old)	Wound area/cm^2^	Wound formation Time (week)
	Male	Female				
Combination group	22	18	3.49 ± 0.77	57 ± 2.9	3.9 ± 0.2	14 ± 0.53
Epidermal growth factor group	20	20	2.99 ± 0.63	59 ± 3.3	4.3 ± 0.1	13 ± 0.62
Nano silver group	19	21	3.01 ± 0.51	56 ± 2.8	4.1 ± 0.3	15 ± 0.17
Control group	18	22	3.51 ± 0.65	60 ± 1.4	4.0 ± 0.3	14 ± 0.35
*p* Value	>0.05	>0.05	>0.05	>0.05	>0.05	>0.05

### Comparison of Wound Healing Effects in Each Group

The time required for wound repair to reach grades 1, 2, and 3 in the epidermal growth factor group were 6.8 ± 0.12, 24.2 ± 0.37, and 35.5 ± 0.27 respectively. In the nano-silver dressing group, the time required for wound repair to reach levels 1, 2, and 3 were 7.1 ± 0.19, 27.8 ± 0.21, and 39.6 ± 0.75 respectively. The time required for the wound repair to reach levels 1, 2, and 3 in the combination group were 6.2 ± 0.32, 19.1 ± 0.24, and 30.3 ± 0.86 respectively. The time required for wound repair to reach grades 1, 2, and 3 in the control group were 7.5 ± 0.13, 30.3 ± 0.19, and 41.5 ± 0.36 respectively.

There was no significant difference in the time required for wound repair to reach grade 1 in the four groups (*p* > 0.05). Compared with the control group, the time required for wound repair to reach grades 2 and 3 was shorter in the combination group and the epidermal growth factor group (*p* < 0.05), and the time required for wound healing in the combination group to reach grades 2 and 3 was shorter than that of the epidermal growth factor group (*p* < 0.05). In the nano-silver dressing group, compared with the control group, there was no significant difference in the time required for wound repair to reach grades 2 and 3 (*p* > 0.05) (Table 2).

**TABLE 2:** Comparison of the time required for wound repair to reach grades 1, 2, and 3 in each group.

Group	40% wound surface healing time/day	70% wound surface healing time/day	Complete wound healing time/day
Nano-silver dressing group	7.1 ± 0.19	27.8 ± 0.21	39.6 ± 0.75
Epidermal growth factor group	6.8 ± 0.12	24.2 ± 0.37*	35.5 ± 0.27*
Combination group	6.2 ± 0.32	19.1 ± 0.24**	30.3 ± 0.46**
Control group	7.5 ± 0.13	30.3 ± 0.19	41.5 ± 0.36

*p < 0.05 vs. control group; **p < 0.01 vs. control group and *p* < 0.05 vs. epidermal growth factor group.

### Comparison of the Growth Rate of New Granulation Tissue in Each Group

The time required for the regeneration of new granulation tissue in the epidermal growth factor group to reach levels 1, 2, and 3 were 5.7 ± 0.53, 22.1 ± 0.55, and 33.2 ± 0.16 respectively. The time required for the repair of newborn granulation tissue in the nano-silver dressing group to reach 1, 2, and 3 were 6.3 ± 0.15, 25.9 ± 0.36, and 37.1 ± 0.23 respectively. The time required for the new granulation tissue repair to reach grades 1, 2, and 3 in the combination group were 5.5 ± 0.31, 17.3 ± 0.27, and 26.3 ± 0.58 respectively. The time required for the repair of newborn granulation tissue in the control group to reach 1, 2, and 3 were 6.7 ± 0.13, 28.7 ± 0.24, and 40.1 ± 0.15 respectively.

There was no significant difference in the time required for the repair of newborn granulation tissue to reach grade 1 in the four groups of patients (*p* > 0.05). Compared with the control group, the time required for the new granulation tissue repair to reach grades 2 and 3 was shorter in the combination group and the epidermal growth factor group, the difference was statistically significant (*p* < 0.05); and the time required to repair the new granulation tissue to grades 2 and 3 in the combination group was shorter than that of the epidermal growth factor group (*p* < 0.05). Compared with the control group, there was no significant difference in the time required for the repair of newborn granulation tissue to reach grades 2 and 3 in the nano-silver dressing group (*p* > 0.05) (Table 3).

**TABLE 3:** Comparison of the time required for the regeneration of new granulation tissue reach grades 1, 2, and 3 in each group.

Group	Time need for grade 1/day	Time need for grade 2/day	Time need for grade 3/day
Nano-silver dressing group	6.3 ± 0.15	25.9 ± 0.36	37.1 ± 0.23
Epidermal growth factor group	5.7 ± 0.53	22.1 ± 0.55*	33.2 ± 0.16*
Combination group	5.5 ± 0.31	17.3 ± 0.27**	26.3 ± 0.58**
Control group	6.7 ± 0.13	28.7 ± 0.24	40.1 ± 0.15

*p < 0.05 vs. control group; **p < 0.01 vs. control group and *p* < 0.05 vs. epidermal growth factor group.

### Results of Bacterial Culture in Four Groups of Patients

The positive bacterial culture in the nano-silver dressing group and combination group was significantly lower than that in the control group both in 2 and 4 weeks after treatment, and the difference was statistically significant (*p* < 0.05). In the epidermal growth factor group, the positive rate of bacterial culture of wound exudate was not significantly different from the control group both in 2 and 4 weeks after treatment. The specific results are shown in Table 4. The bacterial count of each treatment stage: there was no significant difference in the bacterial count in the original wound exudate between the groups in the initial period (grade 0). In grades 1, 2, and 3, the bacterial count level of the combined group and the silver nanoparticles group were all lower than that of the control group, and there was no significant difference between the bacterial count of the epidermal growth factor group and the control group. The measurement results of the specific bacterial count at each grade were shown in Table 5.

**TABLE 4:** Positive rate of bacterial culture in wound exudate after treatment in each groups.

Group	2 weeks			4 weeks		
	Case	Positive bacterial culture	Positive rate of bacterial culture [% (n/n)]	Case	Positive bacterial culture	Positive rate of bacterial culture [% (n/n)]
Combination group	40	3*	7.5*	38	4*	10.0*
Epidermal growth factor group	40	10	25.0	40	12	30.0
Nano silver group	40	4*	10.0	40	5*	12.5*
Control group	40	9	22.5	40	13	32.5
*p* Value	—	—	<0.05	>0.05	—	<0.05

*p < 0.05 vs control group.

**TABLE 5:** Bacterial counts over time at each level of treatment.

Group/bacterial counts of different stage (10^4 cfu/g)	Grade 0	Grade 1	Grade 2	Grade 3	*p* value
Combination group	4.32	3.15*	2.20**	2.10**	<0.05
Epidermal growth factor group	4.6	4.55	4.45	4.3	>0.05
Nano silver group	4.25	3.10*	2.25**	2.15**	<0.05
Control group	4.45	4.35	4.3	4.2	>0.05
*p* Value	>0.05	<0.05	<0.05	>0.05	—

*p < 0.05 vs. control group, **p < 0.01 vs. control group.

## Discussion

The impact of diabetes on the human body is most directly reflected in the diversity, refractory, and repetitive nature of diabetic complications. Common clinical complications of diabetes include diabetic foot, diabetic neuropathy, and diabetic nephropathy (Guo et al., 2015). Diabetic foot is one of the main complications of diabetic patients. It is caused by abnormal metabolism of diabetes, lower limb neurological dysfunction, peripheral vascular structural abnormalities which leads to foot deformation, ulcer formation, and gangrene of the extremities (Betsch et al., 2018). Diabetic patients have a low immune function, and the ulcer-producing foot is susceptible to infection, difficult to heal, and has a poor prognosis. The main clinical manifestations are symptoms of lower limb infection, ulcer formation, and/or deep tissue destruction (Sadati et al., 2018), which seriously affect the health of patients and increase the burden on families and society. Therefore, how to effectively treat the disease has important clinical value. At present, the key of treatment for diabetic foot is focused on improving the blood supply to the lower extremities (Al Sayah et al., 2015) and the treatment of diabetic foot ulcers (Binyet et al., 1994). For the diabetic foot in the distal part of the limb, small wounds are generally treated with a topical drug against infection (Xu et al., 2013) or covered with a biological covering (Yang et al., 2012) to promote wound healing.

Chronic refractory wounds are wounds that cannot heal for more than 4 weeks. Diabetic ulcers are the most common, and severe cases can lead to amputation. Related literature shows that (Ou, 2010) 5–15% of patients with diabetes have foot ulcers. The incidence of ulcer amputation is about 50% and the wounds caused by various traumas in diabetic patients can increase amputation rate and mortality. It has a huge impact on the quality of life of patients. Clinical treatment is mostly based on comprehensive treatment, but it requires huge medical resources. Therefore, finding an effective means to promote the healing of such chronic wounds is an urgent social problem.

The wound healing process is complex, including three major stages: inflammation, cell proliferation, and wound remodeling. Complete re-epithelialization and angiogenesis of the wound are the main biological events in the cell proliferation stage. Wetting the wound environment, removing wound bacteria, necrotic tissue and cellular negative can promote wound repair and accelerate healing. Several studies have shown that (Cai et al., 2011) chronic refractory wounds have low levels of EGF or decreased activity, and diabetic dysfunction wounds are affected by abnormal glucose metabolism, “toxic effects” of high glucose and wound repair cells, glycation end products, wound tissue, cell dysfunction, etc., which is also an important reason for the wound healing. Epidermal growth factor widely present in normal human body fluids is an important cytokine that promotes wound repair of skin and mucous membranes. It promotes cell mitosis by promoting the synthesis of DNA, RNA, and hydroxyproline in epidermal cells. In the process of wound healing, epidermal growth factor can promote the proliferation and migration of vascular endothelial cells, the production of fibronectin, the production of fibroblasts, and thus facilitate the growth of granulation of wounds and the proliferation of epithelial cells (Tiaka et al., 2012; Wang, 2013). It plays an important role in regulating dermal and vascular microcirculation and establishing capillary networks. In addition, with the continuous development of scientific research techniques, it has been found that epidermal growth factor is extremely important for improving wound inflammation, inducing inflammatory cell metastasis, and improving the nutritional status of skin tissues, mainly because epidermal growth factors can stimulate the body, and the formation of fibrin shortens the wound healing time in diabetic foot patients (Valenzuela-Silva et al., 2013; Liu et al., 2014).

Nano-silver material is a new type of non-antibiotic antibacterial silver preparation. Its mechanism of action is different from that of chemical antibacterial drugs. It mainly blocks the key enzymes of cell respiration through the passage of silver ions on the bacterial cell membrane and hinders the synthesis of RNA and DNA, and destroys the function of bacterial proteins. It has a broad-spectrum bactericidal effect and is not susceptible to drug resistance. Nano-silver dressings use nanotechnology to prepare nano-silver ultrafine particles. The particles are very small. Compared with ordinary silver dressings, the contact surface is enlarged, and the bactericidal effect is obviously enhanced compared with ordinary silver dressings (Zhang et al., 2015); for *Pseudomonas aeruginosa*, *Staphylococcus aureus*, *streptococcus*, and other types of bacteria have a good inhibitory effect, and will not cause secondary damage to the skin, mainly because the nano silver dressing directly acts on the bacteria. When the bacteria lose their activity, on the one hand, silver the ions will be freed from the cells, to avoid secondary damage to the wound surface. On the other hand, at the same time sustainable release of nano-silver ions, to maintain the effective silver ion concentration of the wound, can play a role in repeated sterilization of the wound surface. Huang Lingyi (Huang et al., 2012) and other combined use of epidermal growth factor and silver ion dressing in diabetic foot patients. The total effective rate of the observation group was as high as 99.8%; the patient’s healing time was significantly shortened, and the number of dressing changes was significantly reduced. This study fully demonstrates the efficacy of epidermal growth factor combined with silver ion dressings in the treatment of diabetic foot. Moreover, nano-silver materials have strong osmotic effects, and no reports of obvious side effects have been found (Betsch et al., 2018).

The results of this study showed that the therapeutic efficacy and wound healing rate of patients in the combination group were better than other groups, indicating that the EGF + nano silver material was an effective treatment for diabetic foot and promoted wound healing. The nano silver material inhibits and kills local pathogenic bacteria, locally exogenous EGF, compensates for the drawbacks of local growth factor deficiency, and synergistically achieves the effect of promoting wound healing. In the epidermal growth factor group, the time required for the wound to heal to the grade 2, 3 was shorter than those of the control group, indicating that the epidermal growth factor alone can also promote wound healing. In the epidermal growth factor group, the positive rate of bacterial culture of wound exudate was similar to that of the control group, which was significantly higher than that of the combined group and the nano-silver dressing group. These suggested that epidermal growth factor had no significant effect on anti-infection, and its main function was to promote wound healing. In the nano-silver dressing group, the time required for the wound to heal to the grade 2, 3 was shorter than those of the control group, but the difference was not statistically significant. The positive rate of the bacterial culture of wound exudate was significantly lower than that of the control group. It is indicated that nano silver mainly exerts an antibacterial effect on wounds and may provide a favorable microenvironment for wound healing by reducing infection and inflammatory response. In the combination group, the time required for the wound to reach the second and third grades was shorter than that of the epidermal growth factor group, which also proved that the combined application of the two had a greater effect on wound healing.

This study used insulin to regulate blood glucose levels, block or reduce the basis of bacterial reproduction, and reduce the incidence of local infection, to avoid the patient’s use of antibiotics due to serious infections affecting the results of the study.

EGF has the characteristics of network regulation of the healing process. Its effect on promoting wound repair may be affected by microenvironment and inflammatory factors. At the same time, the nano silver material was given to completely remove the wound bacteria, reduce the local inflammatory response and provide a better microenvironment for wound healing. The combined use of the two can jointly promote the wound repair and shorten the wound healing time. In this study, 40 of 160 patients were treated with nano silver dressings combined with epidermal growth factors for the treatment of diabetic foot wounds. The effect on wound repair and antibacterial effect was superior to the other groups. When using nano silver dressing combined with epidermal growth factors, attention should be paid to the exudation of the patient’s wound. Although dry wounds are less prone to infection and may be beneficial for healing, if there is less exudation, saline should be added to promote the release of silver ions and enhance the therapeutic effect.

## Conclusion

In conclusion, nano-silver dressing combined with epidermal growth factor for the treatment of diabetic foot can inhibit bacterial proliferation, better control wound infection, accelerate epidermal growth and granulation tissue formation, and promote wound healing. It is an ideal method for treating diabetic foot which is worthy of being promoted in clinical practice.

## Data Availability

The raw data supporting the conclusion of this article will be made available by the authors, without undue reservation, to any qualified researcher.
